# Supplementary material for: Transdermal Drug Delivery in the Pig Skin
Source: Pharmaceutics. 2021 Nov 26;13(12):2016. doi: 10.3390/pharmaceutics13122016 (PMC8707795; doi:10.3390/pharmaceutics13122016)
Supplement: Supplementary file 1 [file pharmaceutics-13-02016-s001.zip › pharmaceutics-1450167-supplementary.pdf]

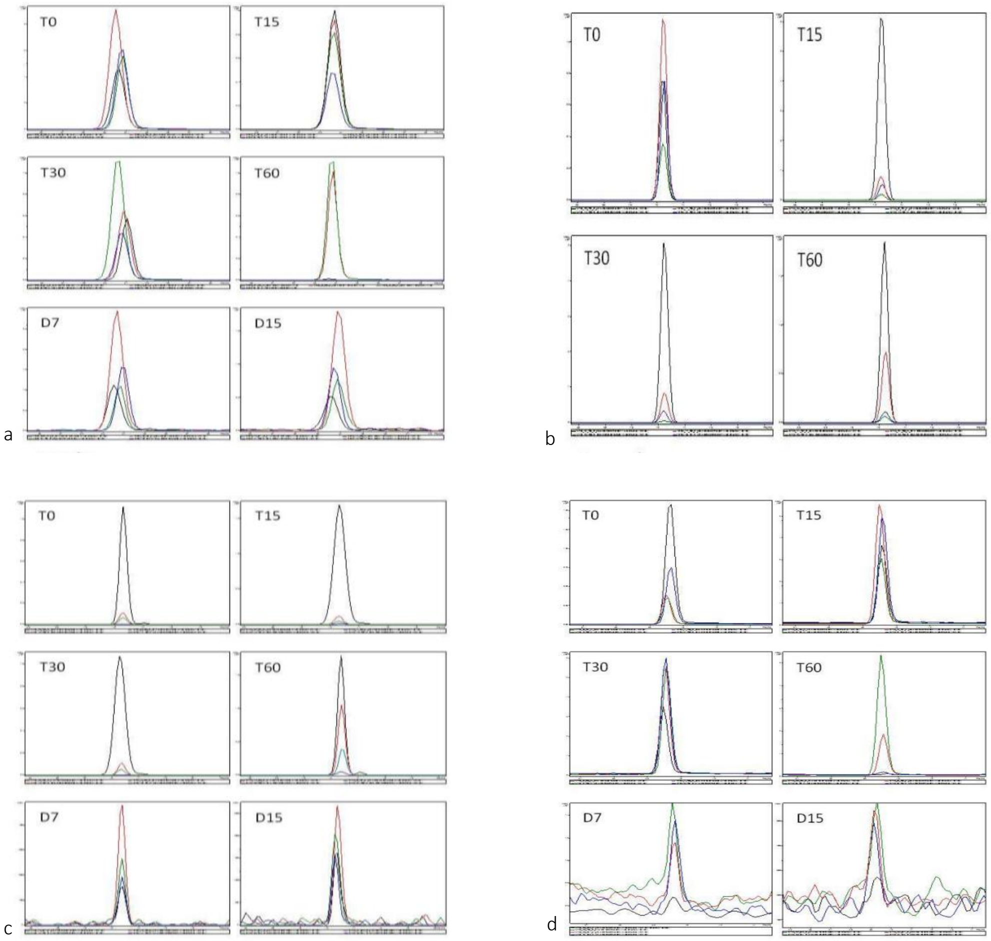

Supplementary Figure S1: HPLC curves for procaine (a), ketoprofen (b), biotin (c) and caffeine (d).

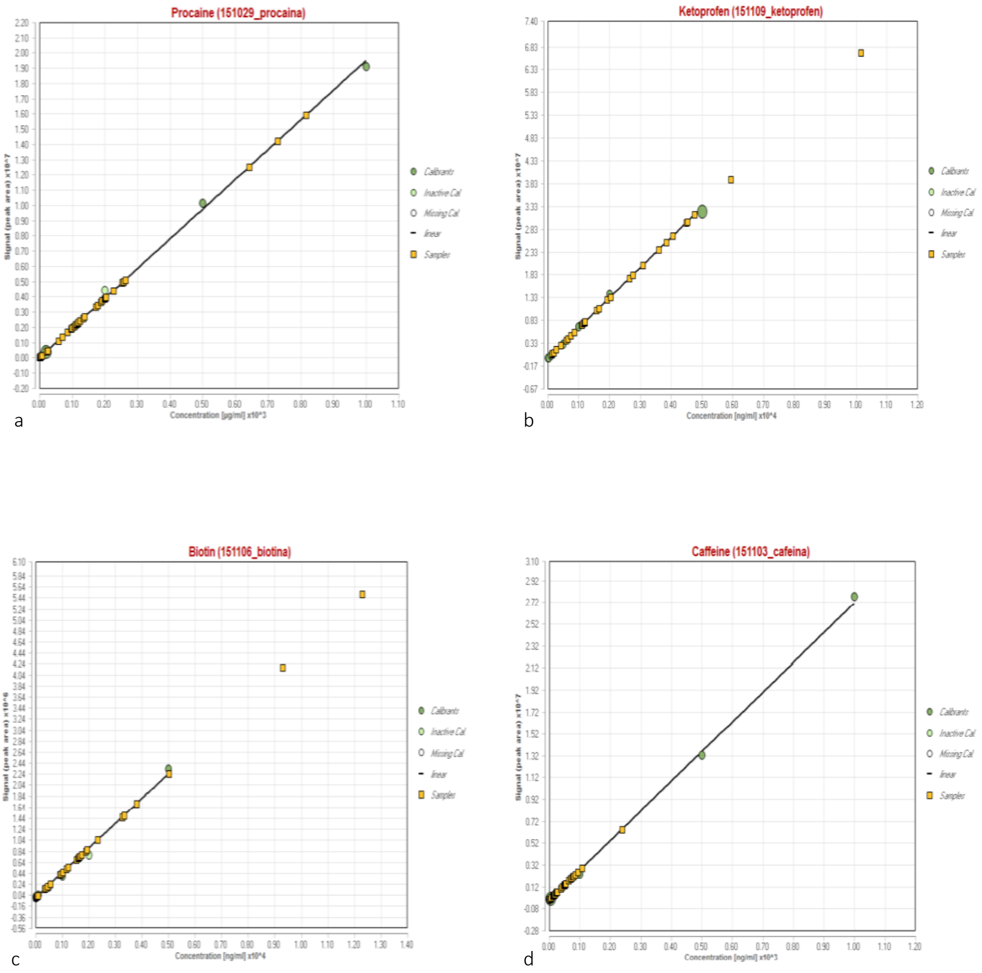

Supplementary Figure S2: calibration values for procaine (a), ketoprofen (b), biotin (c) and caffeine (d).
